# Ontoserver: a syndicated terminology server

**DOI:** 10.1186/s13326-018-0191-z

**Published:** 2018-09-17

**Authors:** Alejandro Metke-Jimenez, Jim Steel, David Hansen, Michael Lawley

**Affiliations:** The Australian e-Health Research Centre, CSIRO, Level 5 UQ Health Sciences Building, Royal Brisbane and Women’s Hospital, Herston, QLD 4029 Australia

**Keywords:** Clinical terminologies, Interoperability, SNOMED CT, FHIR

## Abstract

**Background:**

Even though several high-quality clinical terminologies, such as SNOMED CT and LOINC, are readily available, uptake in clinical systems has been slow and many continue to capture information in plain text or using custom terminologies. This paper discusses some of the challenges behind this slow uptake and describes a clinical terminology server implementation that aims to overcome these obstacles and contribute to the widespread adoption of standardised clinical terminologies.

**Results:**

Ontoserver is a clinical terminology server based on the Fast Health Interoperability Resources (FHIR) standard. Some of its key features include: out-of-the-box support for SNOMED CT, LOINC and OWL ontologies, such as the Human Phenotype Ontology (HPO); a fast, prefix-based search algorithm to ensure users can easily find content and are not discouraged from entering coded data; a syndication mechanism to facilitate keeping terminologies up to date; and a full implementation of SNOMED CT’s Expression Constraint Language (ECL), which enables sophisticated data analytics.

**Conclusions:**

Ontoserver has been designed to overcome some of the challenges that have hindered adoption of standardised clinical terminologies and is used in several organisations throughout Australia. Increasing adoption is an important goal because it will help improve the quality of clinical data, which can lead to better clinical decision support and ultimately to better patient outcomes.

## Background

The problem of sharing and reusing knowledge in software systems is common across many domains. In the area of health there have been several efforts to create clinical ontologies to address this issue, such as SNOMED CT, considered the most comprehensive clinical terminology currently available, with more than 300,000 medical concepts. However, building and maintaining clinical terminologies is considered a hard problem [1, 2]. Despite the availability of SNOMED CT^1^ and other standardised clinical terminologies, many clinical information systems still capture information in plain text or using custom code lists.

There are several challenges that have hindered the widespread adoption of clinical terminologies. The heterogeneity and complexity of specifications results in a significant effort for implementors. For example, SNOMED CT is distributed in *Release Format 2* (RF2) [3], a table-based format that is non-trivial to process (the SNOMED CT implementation guide is over 700 pages long). Other clinical terminologies are modelled in completely different formats. For example, LOINC is distributed in comma-separated values (CSV) files and spreadsheets. There are also many ontologies available natively in OWL format. All of this is compounded by a lack of expertise in the area, which usually requires knowledge in a wide range of technologies, including programming, description logics and ontology reasoners. A terminology server should be able to provide access to any clinical terminology in a standardised manner and thus shield implementers and end users from all of the underlying complexity.

Some terminologies include a vast amount of content which makes finding a specific concept challenging. In order to drive their adoption, it is of fundamental importance to provide a search mechanism that is effective and responsive. It should also be possible to define subsets for specific contexts, for example a subset of concepts for use in an emergency department, in order to improve the quality of the results and reduce the search space.

Another important difficulty when dealing with clinical terminologies is keeping them up to date. Accessing the content of a clinical terminology usually involves an indexing process that can be time consuming and computationally expensive. As releases become more frequent, this can impose a heavy burden on downstream servers. A terminology server should include a distribution mechanism that facilitates keeping clinical terminologies up to date in a straightforward manner.

Finally, when an existing clinical system does not support coded data or uses proprietary code lists, updating it to use a standardised terminology requires a significant amount of effort. Therefore, stakeholders need to be able to clearly see the value of such an investment. Data analytics is one of the benefits of adopting a standardised clinical terminology that generates significant value. Therefore, a terminology server should be able to provide advanced concept querying capabilities.

In this paper we describe Ontoserver [4], a clinical terminology server based on the HL7’s Fast Health Interoperability Resources (FHIR) standard, which was designed to overcome the challenges mentioned before and therefore contribute to the widespread adoption of clinical terminology.

## Implementation

**Fig. 1:**
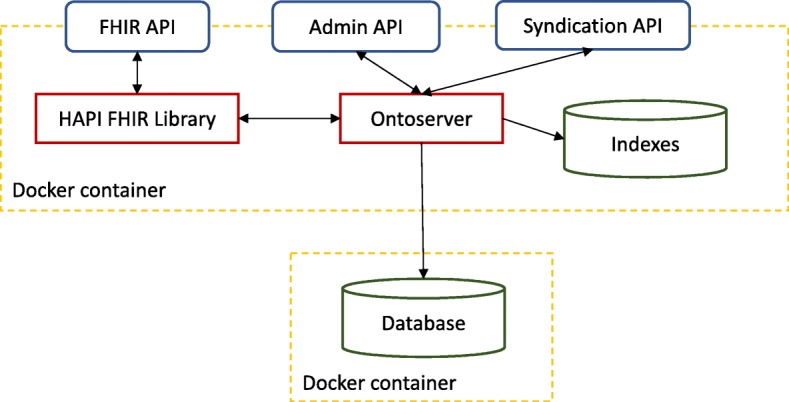
High-level architecture of Ontoserver. Ontoserver is a RESTful server that provides three APIs: a FHIR API, implemented using the HAPI FHIR library, to support terminology functionality, an administration API used to implement functionality that is not defined in the FHIR specification, such as uploading a SNOMED CT code system in RF2 format, and a syndication API that is used to consume and expose syndicated terminology resources. It uses a Postgres database to store FHIR resources and a Lucene index to support searching. The application is deployed using Docker

Ontoserver is implemented as a Java application and its high level architecture is shown in Fig. 1. There are four main features that drove the development of this version of Ontoserver: adopting a terminology service standard, supporting several key clinical terminologies out of the box, designing a mechanism to easily keep the terminologies up to date and providing effective concept search. The following sections describe the implementation of these features in detail.

### Terminology service standard

Ontoserver was implemented based on the terminology subset of the Fast Health Interoperability Resources (FHIR) standard [5, 6]. Other standards, such as the Common Terminology Services 2 (CTS2) [7], could have also been used for this purpose, but FHIR was chosen because of its developer-focused development approach. Adopting the FHIR specification allows Ontoserver to provide a unified API to access any clinical terminology, including LOINC, SNOMED CT and any local extensions with bespoke terms, in a simple and well-defined manner. This also allows clients to easily switch to other FHIR-based implementations. The following is a brief overview of the main resources involved in implementing a FHIR terminology server^2^.

A **code system** represents a set of codes from a system. Each clinical terminology of interest is represented by a code system resource within a FHIR server. Ontoserver provides out-of-the-box support for SNOMED CT and LOINC. It also supports any OWL ontology through an external transformation service. Users can also create and upload custom code systems.

The main operations defined by this FHIR resource are **lookup** and **subsumes**. The lookup operation retrieves details about a concept, such as properties and additional labels (designations). The subsumes operation determines what subsumption relationship holds between the specified codes, if any. More details about the code system resource can be found in the FHIR documentation available at http://hl7.org/fhir/codesystem.html.

A **value set** represents a subset of codes drawn from one or more code systems. A code system usually has a canonical value set that represents all of its codes. Value sets can be implicitly or explicitly defined. Implicit value sets may be defined for a specific code system (such as SNOMED CT or LOINC), based on its underlying semantics. Table 1 shows some examples of implicit value sets in SNOMED CT.

**Table 1:** Examples of SNOMED CT implicit value sets

Description	URL
All concepts	http: //snomed.info/sct?fhir_vs
All concepts subsumed by the clinical finding concept (i.e. all clinical findings)	http://snomed.info/sct? fhir_vs=isa/404684003
All concepts that belong to the emergency department reference set	http://snomed.info/sct? fhir_vs=refset/ 171881000036108

Explicit value sets can be defined by using a combination of include and exclude statements. Within these, the users can refer to codes explicitly, by using filters, or by importing other value sets. The FHIR specification defines a set of filters that is common to all code systems. Each code system can also define filters specific to it. For example, the specification defines a filter, **constraint**, for SNOMED CT that uses the Expression Constraint Language (ECL), a language developed specifically for SNOMED CT to define subsets of concepts that satisfy certain criteria [8]. This filter is not used with other code systems because the ECL is specific to SNOMED CT.

The **expand** operation on a value set resolves its members. When a value set is defined as a list of codes, the result of this operation is trivial. However, complex value sets that use filters or import other value sets need to be evaluated at runtime to produce the expansion. The other operation offered by the value set resource is **validate-code** which indicates if a code is part of a value set. More details about the value set resource can be found in the FHIR documentation available at http://hl7.org/fhir/valueset.html.

A **concept map** defines the relationships between two sets of codes, i.e., it defines the relationships between a source and a target value set. One of the most important operations supported by the concept map resource is **translate** which returns a mapping between a code from a source value set to a code in a target value set, if such a mapping exists. The mapping includes the type of equivalence between the codes. More details about the concept map resource can be found in the FHIR documentation at http://hl7.org/fhir/conceptmap.html.

### Clinical terminology support

Ontoserver supports several clinical terminologies out of the box. In order to expose them through the FHIR API, Ontoserver needs to be able to import and index content in each native format. The following sections describe how this support is implemented for SNOMED CT, LOINC and OWL ontologies.

#### SNOMED CT

SNOMED CT is currently distributed as a collection of RF2 files. The set of core files is used to represent concepts, descriptions and relationships, which form the primary content of the distribution. In addition to this, there is also an extensible pattern, referred to as reference sets, that is used to provide additional information. The most important of these additional files is the Module Dependency Reference Set (MDRS), which represents dependencies between different SNOMED CT modules, for example, between the Australian extension and the international version, as well as dependencies between different module versions. All of these files can be uploaded to Ontoserver in order to support importing and indexing multiple versions of SNOMED CT modules.

An extension to the RF2 specification that allows representing concrete domains is used in Australia to model the Australian Medicines Terminology. Concrete domains allow modelling properties that have concrete values, such as strings or integers, using predicates such as = or ≤. SNOMED CT was originally built using a subset of the OWL EL profile [9] that did not include concrete domains. Even though most of its content can be correctly modelled using this subset, some concepts cannot be fully modelled without concrete domains. For example, it is impossible to fully represent a *Hydrochlorothiazide 50mg tablet* without using a data literal to represent the quantity of the active ingredient. To overcome this limitation, the Australian Digital Health Agency (ADHA) proposed a mechanism to represent a limited form of concrete domains using RF2 reference sets. This mechanism allows maintaining compatibility with existing RF2 processing tools that do not support concrete domains but adds significant complexity for developers that want to support the new functionality in their tools. Ontoserver also supports this extension.

SNOMED CT also defines the Expression Constraint Language (ECL) which allows building expressions that select sets of concepts. FHIR defines a mechanism to use ECL expressions as filters in value set definitions and the search operators also allow referring to value set membership. Therefore, when systems have data coded with SNOMED CT, ECL can be used to create complex queries through the use of value sets. Ontoserver provides a complete implementation of ECL. More details about the features available for the SNOMED CT code system can be found in the FHIR documentation at http://hl7.org/fhir/snomedct.html.

#### LOINC

LOINC is a database and universal set of test identifiers for medical laboratory observations and other health data [10]. It is currently distributed as a collection of CSV files and spreadsheets packaged in a ZIP file. The LOINC importer extracts the required files from the archive and the LOINC indexer reads them and creates a Lucene index. Each version of LOINC is distributed as a separate archive, so the handling of versions is much simpler than with SNOMED CT. More details about the features available for the LOINC code system can be found in the FHIR documentation at http://hl7.org/fhir/loinc.html.

#### OWL ontologies

OWL ontologies are supported by providing an external service that transforms OWL files into FHIR code system resources. Implementing an external transformation has several advantages over implementing a custom FHIR importer. First, even though a set of sensible defaults needs to be defined for the result of the transformation, the resulting code systems can be easily tweaked by the users before uploading them to the server. Also, an external transformation allows storing the resulting code system in any capable FHIR server, not just Ontoserver. Note that an external transformation service is not suitable for ontologies such as SNOMED CT because these include elements such as implicit value sets that are impractical to create externally.

**Fig. 2:**
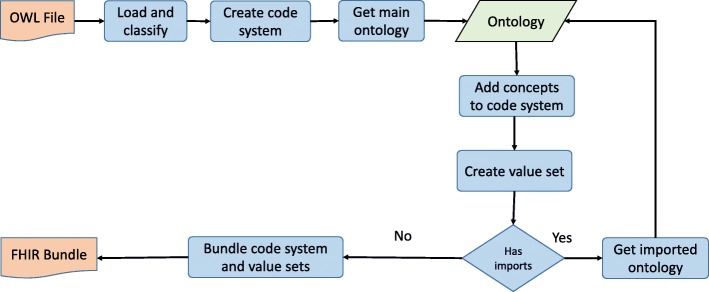
High level view of OWL to FHIR transformation. OWL ontologies are supported in Ontoserver through a service that transforms an OWL file into a FHIR Code System. The process involves classifying the ontology, merging all imported ontologies into a single code system and creating value sets for all imported ontologies

Figure 2 shows the high level steps involved in this transformation. First, the OWL ontology is loaded and classified using any ontology reasoner that supports the OWL-API [11]. Our implementation supports the OWL EL profile but other profiles are supported as long as a reasoner for that profile exists and implements the OWL-API. Then, a FHIR code system is created and its attributes are populated based on certain values in the ontology. These values are shown in detail in Table 2. Most of these are straightforward mappings between attributes in the source ontology and attributes in the target code system. However, the system provides configuration options to override these default mappings when more than one source attribute can be used.

**Table 2:** Code system elements and their corresponding elements in OWL ontologies

Code System	OWL	Comments
Id	-	The id of the resource is local so it can be passed in as a parameter to the transformation.
Url	Ontology IRI	The IRI is optional in an OWL ontology. If it is not present then the transformation stops.
Version	Ontology version	If the ontology has no version, then the version is set to ’NA’. The user can modify this or remove the version altogether.
Name	rdfs:label, Ontology IRI	If the ontology has been annotated with an RDFS label, then the first occurrence is used as the code system’s name. Otherwise the ontology’s IRI is used.
Publisher	Publisher	This element can be configured (the default value is http://purl. org/dc/elements/1.1/publisher).
Description	Subject, rdfs:comment	These elements can be configured (defaults values are http://purl. org/dc/elements/1.1/subject and http://www.w3.org/2000/01/ rdf-schema#comment).
Status	-	Always set to ACTIVE.
ValueSet	Ontology IRI	Set by default to the same URI as the code system.
HierarchyMeaning	-	Always set to SUBSUMES.

The generated code system also includes a set of properties that are mainly used to provide an easy way for clients to access some of the most important information about the concepts. Table 3 shows these properties and how their values are calculated.

**Table 3:** Properties added to the code system

Code System	OWL	Comments
Parent	-	This property is used to provide an easy way for clients to access the direct parents of a concept. This is useful when creating a graphical view of the code system. It is calculated using the reasoner.
Root	-	This property is used to inform clients if a concept is a root. It is calculated using the reasoner.
Deprecated	Annotation	In OWL a class is marked as deprecated by annotating it with a deprecation annotation.

The main challenge in this process is the mismatch in terms of modularity supported by OWL ontologies and FHIR code systems. FHIR code systems do not support modularity, while OWL ontologies support it through an import mechanism. Therefore, once the target code system is created, the system iterates over all the ontologies referenced by the main ontology, including itself. For example, the Human Phenotype Ontology (HPO) imports 11 additional ontologies, some of which also import other ontologies, so this process would iterate over 12 different ontologies, HPO and the 11 additional ontologies referenced by it. All the concepts from every ontology in the imports closure are added to the target code system.

In addition to this, a value set is created for the main ontology (excluding its imports) and for every imported ontology. This allows preserving the correct hierarchy in the code system (because some elements in the hierarchy might come from the imported ontologies) and at the same time allows restricting search to just the main ontology (or any of the imported ontologies) by using the value sets.

### Effective concept search

Finding a concept in a clinical terminology is a key operation in many contexts, such as entering coded data in a clinical system. In many cases entering structured data is an additional burden on the end user, who would typically prefer to capture information using natural language. Therefore it is important that users are able to quickly locate a concept of interest with minimal effort which means the system should return a good ranking of potential matches and should also do it quickly. The use of value sets is useful in this context because it constrains the search to a more manageable set. However, in many cases the search space can still be very large. Also, users are unlikely to formulate entire queries as they would do in a web search scenario but rather just start typing and expect autocomplete-style results.

There have been few studies on this type of search on large clinical terminologies. The most relevant one is the work by Sevenster, van Ommering and Qian [12], where a user experiment was conducted to evaluate two autocompletion algorithms, standard breadth-first (SBF) and multi-prefix matching (MPM), on a large medical vocabulary. The former extends the string the user is typing to the right. For example, if the user searches for *acute append* in SNOMED CT, the algorithm could yield the strings *acute appendicitis* and *acute appendicitis with peritonitis*, for example, but not the string *acute focal appendicitis*. The latter matches the terms whose prefixes match the string typed by the user. For example, the search string *ac app* would match *acute appendicitis*, *acute appendicitis with peritonitis* and *acute focal appendicitis*. The user experiment concluded that the MPM algorithm performed better, requiring fewer keystrokes to obtain a certain target concept. One of the key aspects of the MPM algorithm is how to rank the matches. The authors propose the following scoring function

1 $$ \sigma(F)=\frac1n\sum_{i=1}^{m}\frac{\vert q_{i}\vert}{\vert F(q_{i})\vert}  $$

where *q*_*i*_ is a query prefix, *F*(*q*_*i*_) is a word in the label that matches the prefix, *m* is the number of query prefixes, and *n* is the number of words in the matching label. Note that the order of the query prefixes has no impact on the ranking score.

Searching for a concept in a clinical terminology corresponds to a value set expansion with a filter parameter in FHIR, which is one of the most important and frequently used operations when implementing search interfaces on top of a FHIR server. The FHIR specification does not mandate how the filters should be interpreted so each server is free to implement this functionality however it sees fit. We implement the MPM algorithm in Ontoserver using Lucene with some slight modifications. These are required because the original algorithm does not handle certain cases properly, such as duplicate prefixes. One example of this is ‘pne pne’, which is a search string that could be reasonable to expect from a user searching for the concept ‘Pneumococcal pneumonia’.

### Clinical terminology maintenance

**Fig. 3:**
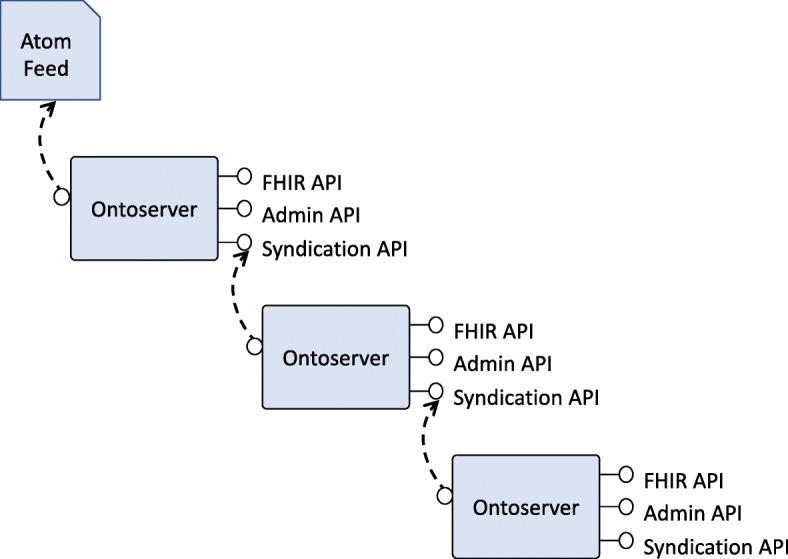
Ontoserver’s syndication model. Ontoserver can consume and publish terminology resources to syndicate, allowing the creation of a syndication chain. In Australia, the Australian Digital Health Agency published an Atom feed that can be used as the first node in the chain

One of the key features of Ontoserver is its ability to act as a syndication client and server. The syndication functionality is implemented using the Atom standard [13]. Figure 3 shows how Ontoserver can act both as a client and a server and therefore create a chain of syndication servers. The idea behind the syndication functionality is to provide an easy mechanism for terminology servers to keep up to date with new releases of clinical terminologies. In Australia, for example, the Australian Digital Health Agency (ADHA) maintains an Atom feed that contains all the releases of SNOMED CT-AU, the local version of SNOMED CT. When an instance of Ontoserver is configured to point at this feed, it can easily check if a new version of SNOMED CT-AU has been published and in that case retrieve it and install it locally.

If an organisation produces its own version of a clinical terminology, for example, an extension of SNOMED CT-AU, they can use their instance of Ontoserver to distribute it downstream. The syndication API includes operations to turn an instance of Ontoserver into a syndication server, and allows selecting which content is to be exposed in the Atom feed it generates.

The syndication mechanism supports both source files and Ontoserver binary indexes. The binary index format can only be processed by Ontoserver but the source files are standard RF2 and can be processed by any client. For example, the ADHA consumes the feed to generate a web page that allows downloading the SNOMED CT-AU releases by a human user.

When a user requests to index a code system in Ontoserver, the system first looks for a compatible binary index in the syndication feed. If it doesn’t find one, then it looks for the source files and builds the index locally. If the source files are also unavailable then the request fails. This functionality was designed in this manner because building an index locally can be computationally expensive and therefore it is preferable to download a prebuilt binary index if available.

## Results and discussion

**Fig. 4:**
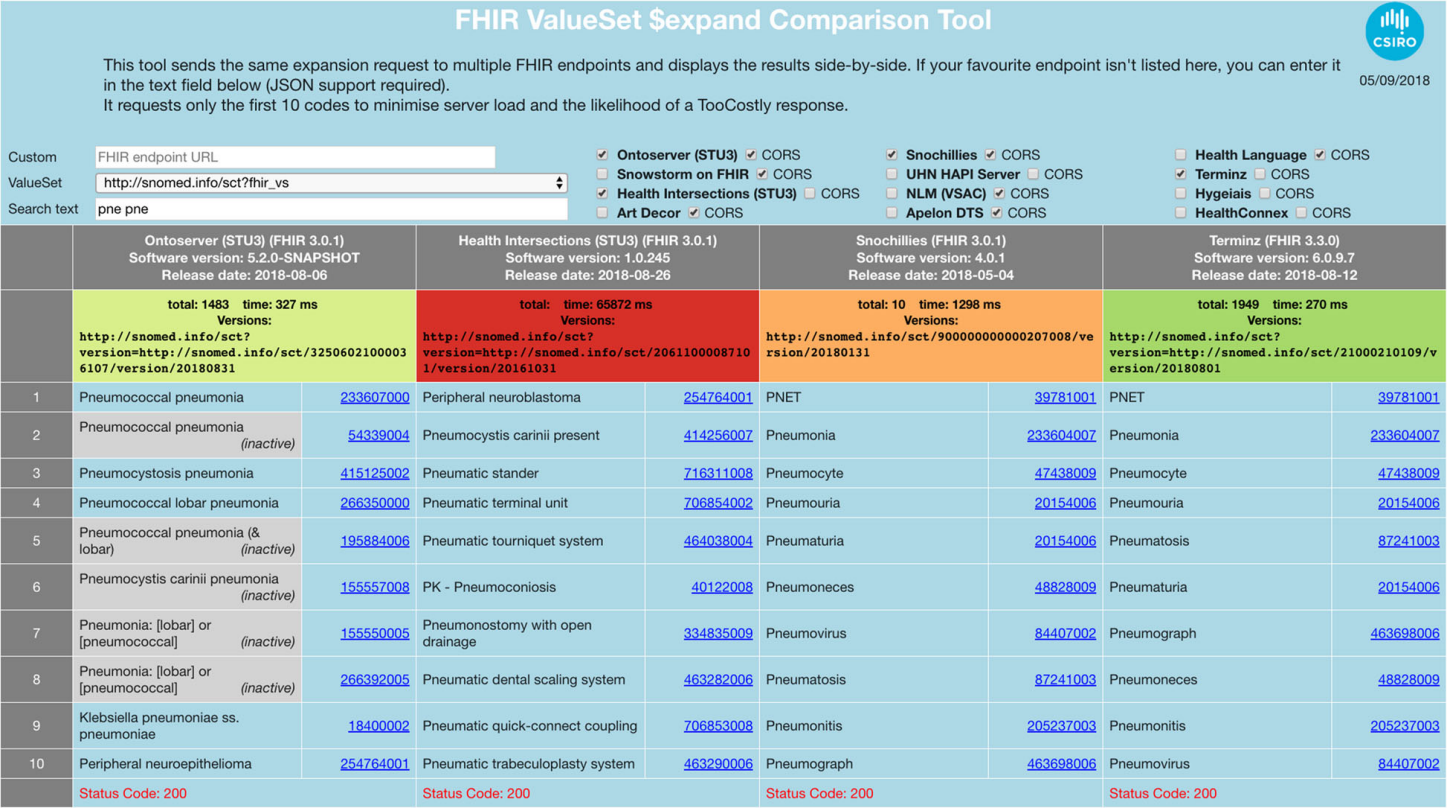
The FHIR ValueSet $expand Comparison Tool. The VSTool can be used to interactively compare the results and response times of different FHIR terminology servers

As mentioned in the “Effective concept search” section, the search algorithm implemented by Ontoserver is a modified version of an algorithm available in the published literature [12] that has already been evaluated against other algorithms in terms of quality of the search results. The modifications only deal with edge cases and therefore the conclusions from the user study are still valid. Another aspect of the evaluation that is important to consider is the performance of the implementation, because the value set expansion operation is likely to be called constantly by user interfaces doing concept search. Having a responsive search widget is essential to providing a good user experience. However, the FHIR specification does not mandate which search algorithm to use. A fair comparison should be performed in practice, balancing speed and success rate, because simpler search algorithms are likely to perform better than more complex ones, but the quality of the search results will be very different. We refer the readers to our VSTool, available at https://ontoserver.csiro.au/vstool/, which allows doing interactive searches across different FHIR terminology servers and looking both at the results returned as well as the response times. Figure 4 shows a screenshot of the tool with the results of the ‘pne pne’ query run across several publicly available FHIR terminology servers, including Ontoserver.

One of the main reasons for the relatively low adoption of clinical terminologies is the perception of stakeholders that the cost of migrating to a standardised clinical terminology might be too high compared to the value it generates. Many terminology servers have been implemented in the past, such as the VOSER vocabulary server [14], the UMLS Knowledge Source Server [15] and the GALEN terminology server [16], among many others. One of the disadvantages of implementing a proprietary terminology server is that clients will have to write different code to interact with it. This is the main advantage of implementing a FHIR-compliant terminology server and is the main reason why Ontoserver was migrated to use the FHIR standard in its latest version. Using the FHIR standard mitigates the cost of adoption and also prevents vendor lock-in because other FHIR-compliant implementations can be used as drop-in replacements.

There are additional challenges that have hindered the adoption of standardised clinical terminologies, including the technical complexity of the formats used by different terminologies, the difficulties in keeping them up to date and the complexities of locating concepts in large terminologies. Ontoserver addresses the technical complexity of terminologies by providing out-of-the-box support for SNOMED CT and LOINC, and also implementing an OWL to FHIR transformation service. To our knowledge, no other FHIR terminology server has implemented support for OWL ontologies.

The clinical domain is far from static and clinical terminologies change often. A key challenge when using a sever is keeping it up to date. This is important because it gives users access to the latest content, which might include new concepts and bug fixes, and also because some licenses impose limitations on the use of older versions. This challenge is addressed by implementing an open syndication mechanism. The National Clinical Terminology Service (NCTS), developed in Australia by the ADHA in collaboration with our research group, developed a syndication API standard^3^ that is implemented by Ontoserver. Having the possibility of acting as both a server and a client for syndication of terminology content enables a straightforward mechanism to maintain multiple instances up to date with the most recent version of the terminologies of interest.

Finding the right concept is also a key challenge, especially when using very large terminologies. This is addressed by implementing a smart search algorithm based on the published literature and modifying it slightly to account for certain edge cases that are present in SNOMED CT.

Once a terminology server is adopted and data is available in coded form, Ontoserver generates value by providing the building blocks for doing more sophisticated analytics. FHIR supports a wide range of search operators that can be used as the basis for this type of functionality, for example, the modifiers **above** and **below** for coded types, which allow users to search not only for a specific code but also for codes that subsume or are subsumed by a code. For example the query:

http://myhost.com/fhir/Observation?code:below=http://snomed.info/sct|118188004

will return all the observations that refer to findings of neonates, that is, all the observations types that are subsumed by the concept *118188004 | Finding of neonate*.

**Fig. 5:**
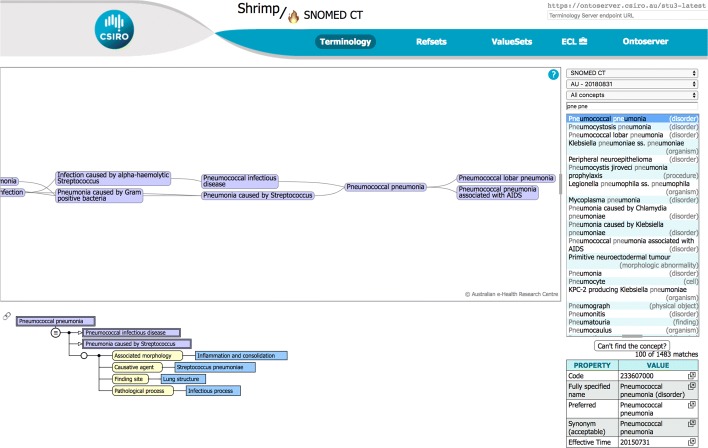
The Shrimp terminology browser Shrimp is a browser. for hierarchical terminologies like SNOMED CT and AMT. It is HTML5, SVG, and Javascript based, and runs in most modern web browsers

Ontoserver is currently in use in several projects. Shrimp is a terminology browser that uses Ontoserver to implement fast concept search and display a graphical view of the search results when the terminology is hierarchical^4^. A screenshot with the ‘pne pne’ search example discussed previously is shown in Fig. 5.

**Fig. 6:**
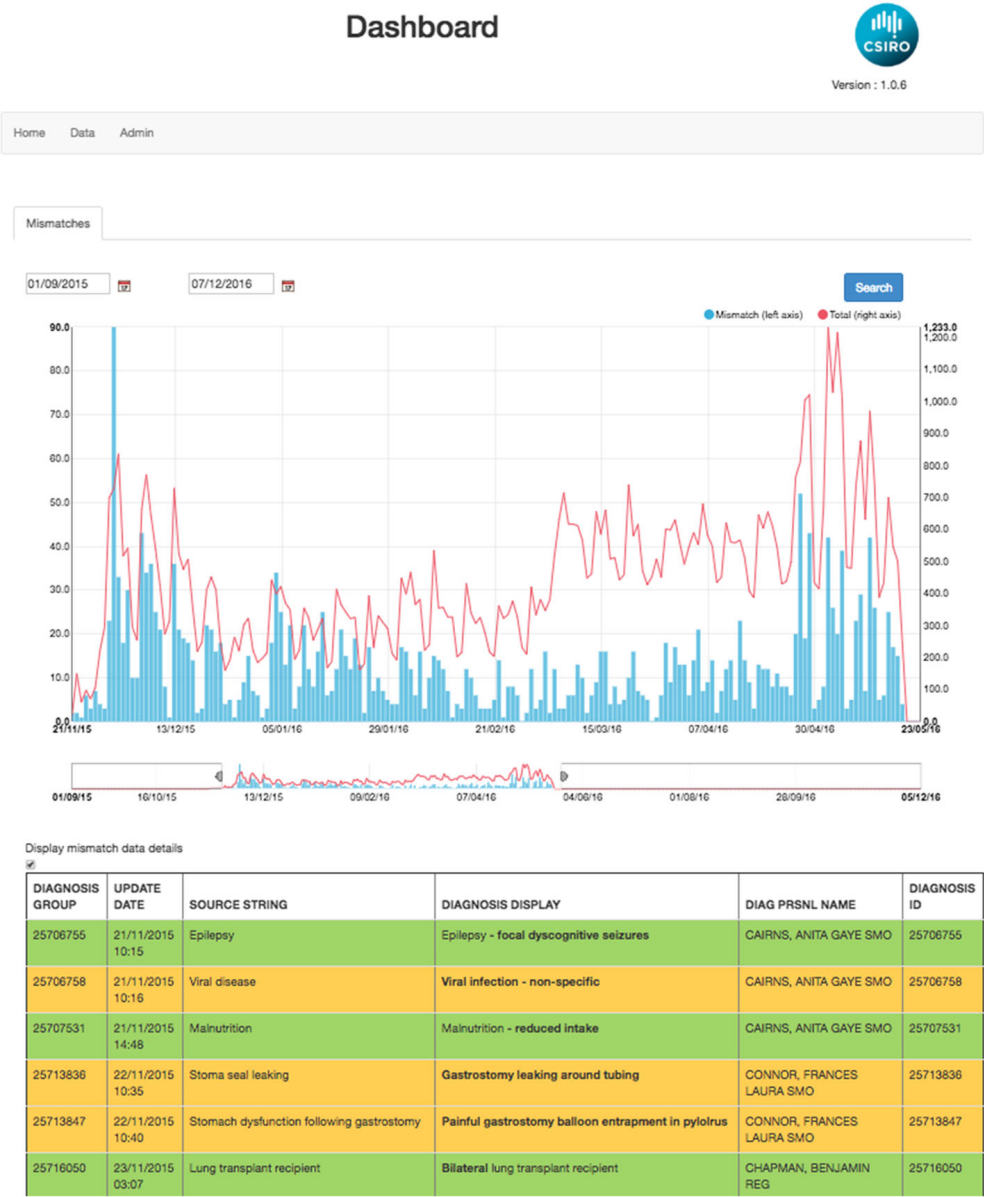
The Escargot data validation tool. Escargot is a web tool that retrieves data from a FHIR server and uses the value set validate-code operation to determine which code descriptions have been modified and no longer match any of the original labels

Escargot is a quality assurance tool that uses Ontoserver’s validation capabilities to find code labels that have been changed by end users and attempts to automatically identify problematic cases. Some clinical systems allow modifying the description of a code once it is entered. This can lead to undesirable user behaviour such as selecting a concept that seems close enough and modifying its display text. This can seriously impact data quality and could even have serious consequences in the context of a clinical decision support system. Escargot retrieves data from a FHIR server and uses the value set **validate-code** operation to determine which code descriptions have been modified and no longer match any of the original labels. It then uses patterns to automatically flag potentially problematic changes. Figure 6 shows a screenshot of the application.

Finally, an example of the use of Ontoserver’s ECL implementation can be found in a tool called SNOMap, which was developed as a way to automatically map SNOMED CT codes into ICD10-AM codes. This is important because many organisations still rely on ICD codes to support Activity Based Funding so migrating a system to use SNOMED CT, which is clinically-focused and more granular, can impact this model. The tool is now in use in several hospitals in Australia.

## Conclusion

Despite the availability of many high-quality clinical terminologies such as SNOMED CT and LOINC, there has been a slow uptake of terminology in clinical systems. In this paper we identify several key challenges that have hindered adoption and show how Ontoserver has been designed to help overcome them. This is important because adoption of standardised clinical terminologies and the use of coded data is key to improving the quality of clinical data, which can lead to better clinical decision support and ultimately to better patient outcomes. We also show examples where Ontoserver is currently being used.

There are three main features that are planned for future versions of Ontoserver. The first one is boosting the ranking of search results based on a value set. This is useful when constraining the results to a value set is too restrictive and it is still desirable to give the user the possibility of selecting a concept from a bigger set. The second one is support for post-coordination. FHIR defines a closure operation that can be used to maintain a client-side closure table based on the terminological logic in the server that is built incrementally. When a client encounters a code and needs to run searches that involve accessing its hierarchy, it requests the data needed to complete the closure table from the server. One of the most interesting features of the closure operation is that it can also work with post-coordinated concepts, i.e., concepts that can be built on the fly if they don’t exist as pre-coordinated concepts in the terminology. Future work will focus on adding post-coordination support to the closure operation, which is currently implemented in Ontoserver but does not support post-coordination. Finally, when building concept maps, it is often necessary to search for potential matches for an entire string, not just a prefix. We refer to this type of search as ‘automap’ because it can be used to produce an initial set of candidates when building a map between two code systems. The current implementation is very simple and future work will explore more sophisticated approaches with the goal of producing much better quality maps automatically.

## Availability of data and materials

Project name: Ontoserver. Project home page: http://ontoserver.csiro.au/. Operating system(s): Platform independent. Programming language: Java. Other requirements: Docker. License: Free to use in Australia - sublicences available from the Australian Digital Health Agency after 1 July 2016. Any restrictions to use by non-academics: Licence required for commercial use outside Australia. A public Ontoserver instance is available worldwide for free for research purposes at https://ontoserver.csiro.au/stu3-latest/.

## Abbreviations

ADHA: Australian digital health agency
API: Application programming interface
CSV: Comma separated values
CTS2: Common terminology services 2
ECL: Expression constraint language
FHIR: Fast health interoperability resources
HPO: Human phenotype ontology
IHTSDO: International health terminology standards development organisation
IRI: Internationalised resource identifier
MDRS: Module dependency reference set
MPM: Multi-prefix matching
OWL: Web ontology language
RDFS: Resource description framework schema
RF2: Release format 2
SBF: Standard breath-first

## Footnotes

1 SNOMED CT is freely available to all members countries of SNOMED International (formerly the International Health Terminology Standards Development Organisation (IHTSDO)).

2 The descriptions in this section correspond to version 3.0.0 of the FHIR specification, which was used by Ontoserver v5.0.0 at the time of writing. FHIR is an evolving standard and some of these resources might have changed in more recent versions. A directory of published versions can be found at http://hl7.org/fhir/directory.html.

3 NCTS Conformant Server Applications Technical Services Specification, which can be found here: https://www.healthterminologies.gov.au/ncts/#/learn

4 Available at https://ontoserver.csiro.au/shrimp-fhir/.

## References

[1] Rector AL (1999). Clinical terminology: Why is it so hard?. Methods Inf Med.

[2] Cimino JJ (1998). Desiderata for controlled medical vocabularies in the twenty-first century. Methods Inf Med.

[3] SNOMED CT Release File Specifications. https://confluence.ihtsdotools.org/display/DOCRELFMT/SNOMED+CT+Release+File+Specifications. Accessed 12 Aug 2017.

[4] Ontoserver. Next Generation Terminology. https://ontoserver.csiro.au. Accessed 12 Aug 2017.

[5] FHIR V3.0.0. http://hl7.org/fhir/. Accessed 12 Aug 2017.

[6] Bender D, Sartipi K. HL7 FHIR: An agile and RESTful approach to healthcare information exchange. In: Proceedings of the 26th IEEE International Symposium on Computer-Based Medical Systems. IEEE: 2013. p. 326–31. https://ieeexplore.ieee.org/xpl/mostRecentIssue.jsp?punumber=6607262.

[7] Common Terminology Services 2 ^TM^ (CTS2 ^TM^. https://www.ncbi.nlm.nih.gov/pmc/issues/160770/. Accessed 12 Aug 2017.

[8] Expression Constraint Language - Specification and Guide. http://snomed.org/ecl. Accessed 12 Aug 2017.

[9] OWL 2 Web Ontology Language Profiles (Second Edition). https://www.w3.org/TR/owl2-profiles/. Accessed 12 Aug 2017.

[10] Forrey AW, Mcdonald CJ, DeMoor G, Huff SM, Leavelle D, Leland D, Fiers T, Charles L, Griffin B, Stalling F (1996). Logical observation identifier names and codes (loinc) database: a public use set of codes and names for electronic reporting of clinical laboratory test results. Clin Chem.

[11] Horridge M, Bechhofer S (2011). The OWL API: A java API for OWL ontologies. Semantic Web.

[12] Sevenster M, van Ommering R, Qian Y (2012). Algorithmic and user study of an autocompletion algorithm on a large medical vocabulary. J Biomed Inform.

[13] The Atom Syndication Format. https://tools.ietf.org/html/rfc4287. Accessed 12 Aug 2017.

[14] Rocha RA, Huff SM, Haug PJ, Warner HR (1994). Designing a controlled medical vocabulary server: the voser project. Comput Biomed Res.

[15] McCray AT, Razi AM, Bangalore AK, Browne AC, Stavri PZ. The UMLS knowledge source server: a versatile internet-based research tool. In: Proceedings of the AMIA Annual Fall Symposium. American Medical Informatics Association: 1996. p. 164. PMC2233094 8947649

[16] Rector A, Solomon W, Nowlan W, Rush T (1995). A terminology server for medical language and medical information systems. Methods Inf Med.

